# Favorable course of previously undiagnosed Methylmalonic Aciduria with Homocystinuria (cblC type) presenting with pulmonary hypertension and aHUS in a young child: a case report

**DOI:** 10.1186/s13052-018-0530-9

**Published:** 2018-08-13

**Authors:** Luciano De Simone, Laura Capirchio, Rosa Maria Roperto, Paola Romagnani, Michele Sacchini, Maria Alice Donati, Maurizio de Martino

**Affiliations:** 1 Pediatric Cardiology Unit, Anna Meyer Children’s University Hospital, Viale Pieraccini, 24, 50139 Florence, Italy; 2 Post-Graduate School of Pediatrics, University of Florence, Anna Meyer Children’s University Hospital, 50139 Florence, Italy; 3 Nephrology and Dialysis Unit, Anna Meyer Children’s University Hospital, 50139 Florence, Italy; 4 Metabolic and Muscular Unit, Neuroscience Department, Anna Meyer Children’s University Hospital, 50139 Florence, Italy; 5 Department of Health Sciences, University of Florence, Anna Meyer Children’s University Hospital, 50139 Florence, Italy

**Keywords:** Pulmonary hypertension, Atypical hemolytic-uremic syndrome, aHUS, Cobalamin C

## Abstract

**Background:**

Cobalamin C (cblC) defect is the most common inborn error of Vitamin B12 metabolism often causing severe neurological, renal, gastrointestinal and hematological symptoms. Onset with pulmonary hypertension (PAH) and atypical hemolytic-uremic syndrome (aHUS) is rare.

**Case presentation:**

We describe the case of a 2-years old child, previously in good health, admitted to the hospital with severe respiratory symptoms, rapid worsening of clinical conditions, O_2_ desaturation and palmo-plantar edema. The patient showed PAH and laboratory findings compatible with aHUS. cblC defect, an inborn error of metabolism, was identified as the cause of all the symptoms described (cardiac, respiratory and renal involvement). Results of neonatal screening for inborn errors of metabolism had been negative.

Administration of IM OHCbl (intramuscular hydroxocobalamin), oral betaine and symptomatic treatment with diuretics and anti-hypertensive systemic and pulmonary drugs induced dramatic improvement of both cardiac and systemic symptoms.

**Conclusions:**

In this case of cblC defect the metabolic treatment completely reverted symptoms of aHUS and PAH. The course was favorable, and the prognosis is what we foresee for the future.

## Background

Cobalamin C defect (cblC) is the most common inborn error of cobalamin metabolism with estimated incidence around 1:100.000 live births [1]. Onset is typically early in life, most prominent presenting with severe neurological impairment like hypotonia, seizures, failure to thrive, irritability and eventually coma, microcephaly or ocular, hematological, renal and gastrointestinal signs. Recently some anecdotal cases have been described, in which pulmonary hypertension (PAH), isolated or combined with atypical Hemolytic-Uremic Syndrome (aHUS) are the leading symptoms.

PAH is defined by right-heart catheterization as a mean pulmonary artery pressure (mPAP) > 25 mmHg. Prognosis of PAH in children is poor, with an average mortality rate of 25% in three years [2].

aHUS is a rare life-threatening disease, linked to unregulated complement activation causing thrombotic microangiopathy (TMA) with multi-organ involvement such as kidneys, brain, lungs, gastrointestinal tract and cardiovascular system.

In this paper we describe the clinical presentation, the diagnostic process, the treatment and follow-up of a young child with aHUS-cblC defect presenting with severe respiratory and renal symptoms, who responded positively to metabolic therapy.

## Case presentation

A 2-year-old male child was admitted to hospital because of worsening one month-long fatigue and loss of appetite. He was born from non-consanguineous healthy parents; pregnancy, delivery and neonatal course were uneventful. Growth, neurological and cognitive development were normal. The extended newborn screening performed in tandem-mass spectrometry was normal: plasma propionylcarnitine was 1.8 micromol/l (normal value < 3.3) and propionylcarnitine/acetylcarnitine ratio was 0.13 (normal value 0.02–0.21). Family history was negative for cardiac or metabolic diseases.

He presented with respiratory rate 60/min, saturation rate of 85% and palmo-plantar edema. Chest X-Ray showed enlargement of the heart shadow and pulmonary interstitial involvement. Soon after, clinical worsening occurred with cyanosis, O_2_ requirement, anasarca and systemic hypertension (140/90 mmHg).

### Cardiac evaluation

**Fig. 1:**
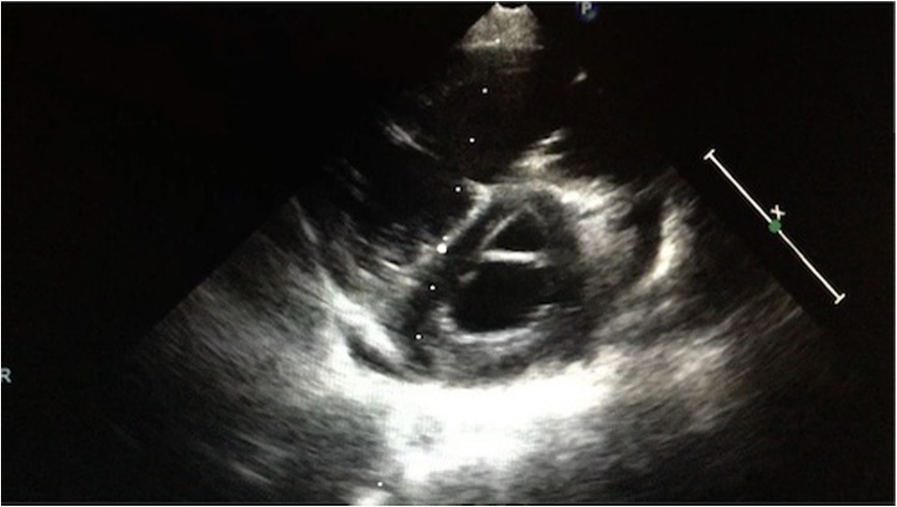
Short axis ventricular view: note straight interventricular septum, typical of right ventricle pressure overload

**Fig. 2:**
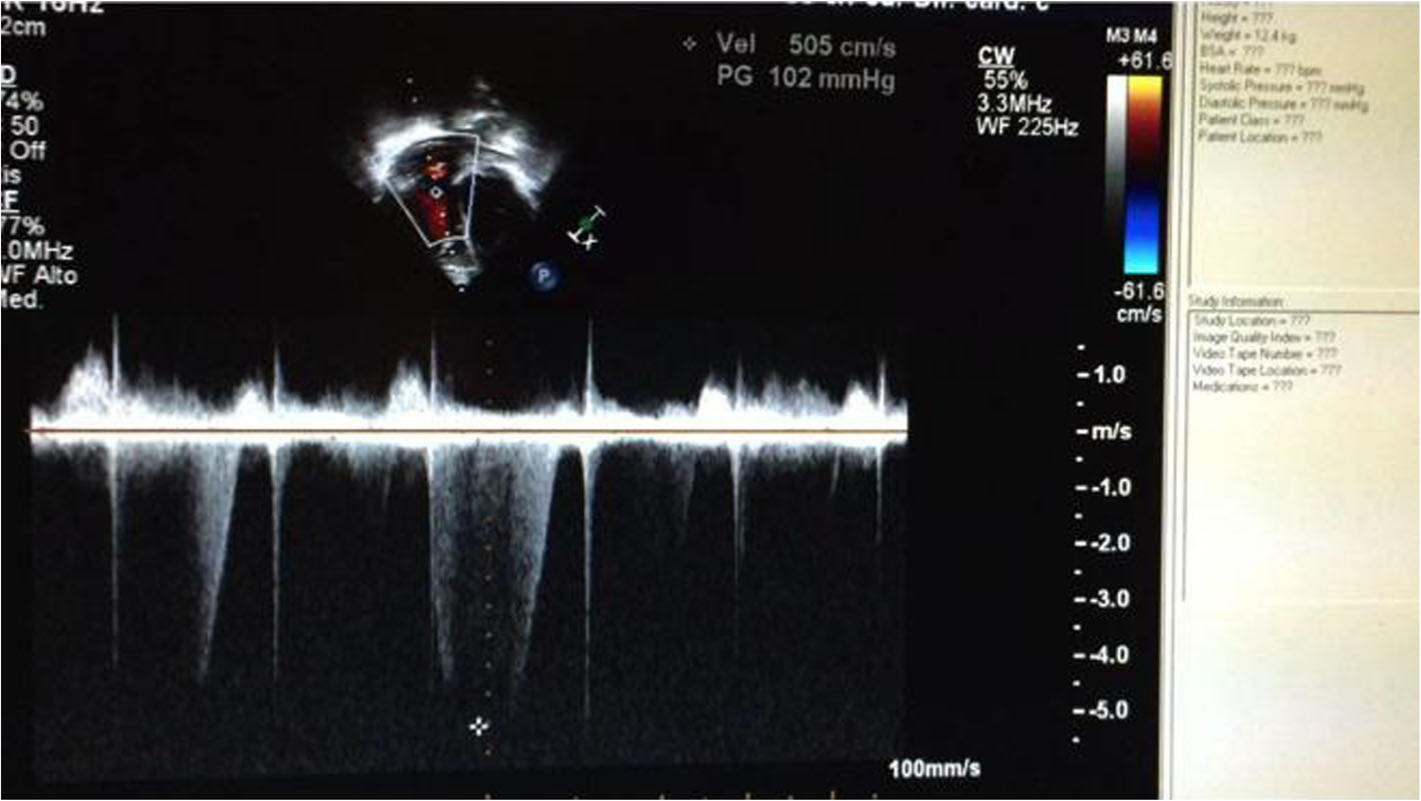
Four-chamber view. CW Doppler evidences high-velocity tricuspid insufficiency: 5.05 m/sec corresponding to pulmonary pressure of 107 mmHg

Right systolic murmur was appreciable. ECG evidenced sinus tachycardia with right ventricular hypertrophy and overload; echocardiogram showed severe right ventricle enlargement, poor ventricular function with paradox movement of interventricular septum (Fig. 1); pulmonary pressure rate, calculated on the basis of tricuspid insufficiency velocity, resulted elevated: 107 mmHg (Fig. 2); left atrium volume was normal and left ventricle resulted hypertrophic. A prompt treatment for both systemic and pulmonary hypertension was started with atenolol/amlodipine and sildenafil, inducing only partial improvement of symptoms.

### Laboratory findings

Blood examinations revealed hemolytic-uremic syndrome (HUS), with macrocytic anemia (hemoglobin 9.0 g/dl, normal values [nv] 10.7–13.4; MCV 86.1 ft., nv 75–85), thrombocytopenia (platelets count 40000 × 103; nv 210–590), elevated LDH (up to 3000 IU/L, nv 192–321) and creatinine (from 0.56 to 1.2 mg/dl, nv 0.2–0.43), low albumin rate (2.6 g/dl, nv 3.5–4.5), very low haptoglobin levels (< 7.5 mg/dl; nv 30–200), negative Coombs test (both direct and indirect), proteinuria and hematuria; complement serum levels resulted low (C3 69 mg/dl, nv 90–180; C4 9 mg/dl, nv 18–55). Hypersegmented neutrophils and schistocytes in the peripheral blood smear were detected.

HUS is one of the most common TMA syndromes. The pathological feature of TMA is vascular damage, manifested by arteriolar and capillary thrombosis with characteristic abnormalities in the endothelium and vessel wall. The most common form of pediatric TMA is the so-called “typical” HUS in which vascular damage is caused by an enteric infection with a Shiga toxin–secreting strain of *Escherichia coli* (STEC) or *Shigella dysenteriae*. Other common forms of TMA are atypical HUS (aHUS) and thrombotic thrombocytopenic purpura (TTP). The latter was excluded because ADAMTS13 activity was normal and autoantibody inhibition of ADAMTS13 activity was absent. Causes of aHUS were examined and excluded except for metabolic diseases. Renal histology was not performed because of severe cardiac involvement of the patient.

### Metabolic evaluation

Workup for inborn error metabolic diseases was started, evidencing: high levels of plasma total homocysteine (tHCy) (74 micromol/l, nv < 15), plasma propionylcarnitine (5.64 micromol/l, nv 0.86), serum methylmalonic acid (138 micromol/l, nv < 1) and urinary methylmalonic acid (919 mM/mol, n.v. < 2); normal value of folic acid and vitamin B12. According to the diagnosis of methylmalonic acidemia and homocystinuria, the child was started on treatment with IM OHCbl (intramuscular hydroxocobalamin) (1 mg/day), oral betaine (250 mg/kg/day), folinic acid (3.75 mg/day) and carnitine (50 mg/kg/day). Response to therapy was dramatic: after 15 days plasma propionylcarnitine was normal (0.74 micromol/l, nv 0.86), serum methylmalonic acid was 0.92 micromol/l (nv < 1), and tHCy was 20 micromol/l (nv < 15).

Molecular analysis of MMACHC gene was performed: cblC defect was confirmed by revealing compound heterozygous c.271-272dupA (frameshift mutation)/c.347 T > C (missense mutation) usually associated with late-onset cblC defect.

### Outcome and follow up

Sildenafil was suspended after 10 days and only a mild anti-hypertensive therapy with enalapril was maintained and suspended after six months. After two years of follow-up, the child is in now good health conditions, showing normal growth, normal mental and neurological development, and no behavioral problems were detected. We performed ocular studies every 6 months and no ocular maculopathy was detected. The current therapy consists in IM OHCbl 1 mg/day, oral betaine (200 mg/kg/day), folinic acid (3.75 mg/day) and carnitine (50 mg/Kg/day). Blood pressure is in normal range; echocardiogram shows normal dimensions and thickness of cardiac chambers, with continent tricuspid valve. Laboratory findings at the last clinical assessment are normal, particularly plasma tHCy (11.6 micromol/l), plasma amino acids and plasma acylcarnitines. Serum methylmalonic acid is 1.5 micromol/l.

## Discussion and conclusions

The patient presented here, affected by cblC defect, showed a prompt response to metabolic therapy and excellent immediate and mid-term outcome. Genetic analysis evidenced compound heterozygosity for 271-272dupA and c.347 T > C genotype on MMACHC gene; it is well known that patients that are compound heterozygotes for a missense allele and c.271dupA appear to have a milder phenotype, suggesting a residual protein function. In late-onset forms it is possible to find negative newborn screening, although a low cut-off for propionylcarnitine is usually used (3.3 micromol/l) and a second tier test (searching for plasmatic methylmalonic acid) is performed [3]. Second-tier testing is performed on newborns with abnormal screening result. It is a second level test performed on the original blood spot in order to find a target analyte under optimum operating conditions. The introduction of second-tier testing, in newborn screening programs, has allowed the reduction of the number of false positives that were resulted in the increase of laboratory analyses. However, as this case shows, the negativity of neonatal screening is not enough to exclude definitively cblC defect until today.

A severe complication of cblC defect is TMA, a typical vascular injury with arteriolar and capillary thrombosis which has been found both in kidneys and lungs of patients affected by aHUS and PAH [4].

Firstly, Van Hove et al. in 2002 reported two siblings with cblC defect and late-onset TMA both presenting renal failure which promptly responded to intensive treatment with hydroxocobalamin and betaine [5].

Only recently PAH has been stressed as an acute complication and a cause of death in the youngest patients affected by aHUS-cblC defect. Indeed, PAH was the first diagnosis performed on our patient based on clinical, radiological and echocardiographic findings. PAH is rare in childhood, mostly confined to the neonate, as persistence of fetal circulation or secondary to congenital heart disease [6, 7]; whether primary or secondary to another disease, it may be a cause of death [2].

In recent ESC Guidelines the classification of PAH mentions some forms secondary to metabolic diseases, but not to Methylmalonic Aciduria with Homocystinuria cblC-type [8] (Table 1).

**Table 1:** Clinical classification of pulmonary hypertension (Simonneau et al. 2013)

1. Pulmonary arterial hypertension 1.1 Idiopathic 1.2 Heritable 1.2.1 BMPR2 mutation 1.2.2 Other mutations 1.3 Drugs and toxins induced 1.4 Associated with: 1.4.1 Connective tissue disease 1.4.3 Portal hypertension 1.4.4 Congenital heart disease 1.4.5 Schistosomiasis 1′. Pulmonary veno-occlusive disease and/or pulmonary capillary haemangiomatosis 1′.1 Idiopathic 1′.2 Heritable 1′.2.1 EIF2AK4 mutation 1′.2.2 Other mutations 1′.3 Drugs, toxins and radiation induced 1′.4 Associated with: 1′.4.1 Connective tissue disease 1′.4.2 HIV infection 1″. Persistent pulmonary hypertension of the newborn	
2. Pulmonary hypertension due to left heart disease 2.1 Left ventricular systolic dysfunction 2.2 Left ventricular diastolic dysfunction 2.3 Valvular disease obstruction and congenital cardiomyopathies 2.5 Congenital /acquired pulmonary veins stenosis	
3. Pulmonary hypertension due to lung diseases and/or hypoxia 3.1 Chronic obstructive pulmonary disease 3.2 Interstitial lung disease 3.3 Other pulmonary diseases with mixed restrictive and obstructive pattern 3.4 Sleep-disordered breathing 3.5 Alveolar hypoventilation disorders 3.6 Chronic exposure to high altitude 3.7 Developmental lung diseases (Web Table III)	
4. Chronic thromboembolic pulmonary hypertension and other pulmonary artery obstructions 4.1 Chronic thromboembolic pulmonary hypertension 4.2 Other pulmonary artery obstructions 4.2.1 Angiosarcoma 4.2.2 Other intravascular tumors 4.2.3 Arteritis 4.2.4 Congenital pulmonary arteries stenoses 4.2.5 Parasites (hydatidosis)	
5. Pulmonary hypertension with unclear and/or multifactorial mechanisms 5.1 Haematological disorders: chronic haemolytic anaemia, myeloproliferative disorders, splenectomy 5.2 Systemic disorders: sarcoidosis, pulmonary histiocytosis, lymphangioleiomyomatosis, neurofibromatosis 5.3 Metabolic disorders: glycogen storage disease, Gaucher disease, thyroid disorders 5.4 Others: pulmonary tumoral thrombothic microangiopathy, osing mediastinitis, chronic renal failure (with/without dialysis), segmental pulmonary hypertension	

Up to now, only few studies in literature report the combination of PAH with aHUS in cblC defect. In 2009, Profitlich et al. first described a 3-year-old child with already known cblC defect presenting with right heart failure and PAH, with complete resolution after aggressive medical management [9]. Compared to this case, our patient had previously been completely asymptomatic before and the negativity of neonatal screening complicated the diagnostic procedure.

The same group reported a retrospective analysis of echocardiographic data of 10 patients affected by cblC defect: five of them presented structural heart defect but only one showed PAH [10].

Kömhoff et al. in 2013 reported a case series of five children with PAH, HUS and cblC deficiency, with disease-onset between 1.5–14 years of age and unfavorable progression [4]. Isolated PAH as main symptom of cblC defect was reported by Iodice et al. in 2013 [11] and Gunduz et al. in 2014 [12], respectively describing two cases of cblC defect with different outcomes despite starting the same vitamin therapy. All those cases presented a genotype correlated with late-onset methylmalonic aciduria and homocystinuria like our child.

A recent study from Beck et al. in 2016 [13] reviewed the biochemical, genetic, clinical and histopathological data from 36 patients affected by renal disease associated with cblC deficit: 2/3 of the patients presented aHUS, 16 underwent renal biopsy showing lesions ascribable to TMA. Cardiac involvement was present in 14 patients, diagnosis of PAH was performed in seven of them, resulting fatal in four.

Recently, Barlas et al. described the case of an infant with aHUS-cblC defect accompanied by complement dysregulation. In this case the pathogenetic mechanism was analyzed because of the non-response to appropriate metabolic therapy. The child was then treated with eculizumab with good results [14].

In conclusion, it is interesting to observe how the same defect may be correlated to different symptoms. The high value of plasma tHCy, impaired methyl-group metabolism and oxidative stress could represent the main pathophysiologic mechanism involved in neurological, cardiovascular and renal complications, but is still not known the cause of the extreme variability of the clinical spectrum, response to therapy and prognosis. The case presented here, however, shows the reversibility of these complications with adequate and prompt therapy.

To the best of our knowledge, this is one of the few cases reported with cblC defect manifesting simultaneously aHUS and PAH in early childhood with favorable outcome and stable over time.

Therefore, in case of a child presenting with PAH and aHUS, we recommend considering cblC defect. This is a treatable congenital defect of cobalamin metabolism in which early specific treatment with IM OHCbl and oral betaine can positively influence the outcome.

## Abbreviations

ADAMTS13: A disintegrin and metalloproteinase with thrombospondin1 motifs (13th member of the family)
aHUS: Atypical hemolytic-uremic syndrome
cblC: cobalamin C
HUS: Hemolytic-uremic syndrome
IM OHCbl: Intramuscular hydroxocobalamin
MMACHC: Methylmalonic aciduria (cobalamin deficiency) cblC type with homocystinuria gene
PAH: Pulmonary arterial hypertension
STEC: Shiga toxin-producing *Escherichia coli*
tHCy: Plasma total homocysteine
TMA: Thrombotic microangiopathy
TTP: Thrombotic thrombocytopenic purpura
